# Reliability and validity of the KIDSCREEN-52 health-related quality of life questionnaire in a Greek adolescent population

**DOI:** 10.1186/1744-859X-11-3

**Published:** 2012-02-13

**Authors:** Chara Tzavara, Anastasia Tzonou, Ioannis Zervas, Ulricke Ravens-Sieberer, Christine Dimitrakaki, Yannis Tountas

**Affiliations:** 1 Centre for Health Services Research, Department of Hygiene, Epidemiology and Medical Statistics, Athens University Medical School, Athens, Greece; 2 Department of Hygiene, Epidemiology and Medical Statistics, Athens University Medical School, Athens, Greece; 3 Department of Psychiatry, Athens University Medical School, Athens, Greece; 4 Robert Koch Institute, Child and Adolescent Health, Berlin, Germany

**Keywords:** adolescents, health-related quality of life, measurement, questionnaire, validity

## Abstract

**Background:**

The KIDSCREEN-52 is a worldwide instrument for measuring health-related quality of life (HRQoL) in children and adolescents. The aim of this study is to assess reliability and validity of the Greek version of KIDSCREEN.

**Methods:**

Questionnaires were collected from a representative nationwide sample of 1,194 adolescents aged from 11 to 17 years. Internal consistency reliability was determined by calculation of the Cronbach α coefficient. A confirmatory factor analysis (CFA) was conducted in order to test the construct validity of the questionnaire. Validity was further examined by investigating the correlation of KIDSCREEN with the Strengths and Difficulties Questionnaire (SDQ) and its association with socioeconomic and health-related factors.

**Results:**

Internal consistency reliability was accepted with a Cronbach α above 0.73 for all KIDSCREEN dimensions. CFA showed that the ten-dimensional model fitted the data well (root mean square error of approximation (RMSEA) = 0.048, comparative fit index (CFI) = 0.971 and goodness of fit index (GFI) = 0.965). Correlation coefficients between KIDSCREEN and SDQ dimensions were significant. Adolescents of low socioeconomic status reported lower scores in the majority of KIDSCREEN dimensions. Also, adolescents with chronic health problem had poorer quality of life concerning physical well-being and other dimensions of KIDSCREEN.

**Conclusions:**

The Greek version of KIDSCREEN-52 was found to have satisfied psychometric properties and could be suitable for assessing HRQoL in Greek adolescents.

## Background

Health-related quality of life (HRQoL) is conceptualized as a multidimensional and comprehensive model of health with several domains. This flows from the definition of health put forward by the World Health Organization (WHO) as a state of complete physical, emotional and social well-being, associated with the individual's perception of their position in life and not the absence of illness [1]. The assessment of HRQoL plays an important role in the assessment of adult health, as indicated by the development of many generic measurement instruments in recent years [2]. The measurement of HRQoL in children and adolescents has received increasing attention in pediatrics and adolescent care and several instruments are now available for use in these groups [3]. Changes in emotional and cognitive development in children and adolescents must be recognized and addressed [4]. Recently, studies have shown that children and adolescents are able to answer the HRQoL questionnaires reliably if their emotional development, cognitive capacity, and reading skills are taken into account [5]. Generic HRQoL instruments can be useful in the identification of groups with health problems or disabilities [6]. Monitoring HRQoL in children and adolescents can also be useful for the evaluation of health services. From an edidemiological perspective it is desirable to have valid HRQoL instruments to aid with public policy decisions and public health promotion strategies and consequently the improvement of population health [6].

The KIDSCREEN-52 52-item questionnaire was funded by the European Commission and measures health-related quality of life of children and adolescents aged 8 to 18 years. The generic KIDSCREEN-52 HRQoL questionnaire is the first instrument for children and adolescents that was developed in several different countries and tested in a large representative sample of children and adolescents [4], thereby helping to provide a broad perspective on the understanding and interpretation of HRQoL across different countries. Psychometric properties such as validity and reliability of the KIDSCREEN-52 HRQoL questionnaire have been assessed in previous studies [7-11] and its crosscultural comparability and psychometric properties have been found satisfactory. The aim of the present study was to assess the reliability and construct validity of the KIDSCREEN-52 quality of life measure in a Greek adolescent population. More specifically, the aim was to examine internal consistency reliability and construct validity by confirmatory factor analysis (CFA) and by the correlation of KIDSCREEN-52 with the scales of the Strengths and Difficulties Questionnaire (SDQ), which investigates emotional and behavioral problems [12]. Also, comparisons according to socioeconomic status and the existence of chronic disease are discussed.

## Methods

### Participants and procedure

The study was conducted during the year 2003 in Greece within the framework of the European project 'Screening and Promotion for HRQoL in Children and Adolescents - A European Public Health Perspective' [4]. The sampling was random, multistaged and based on the age and sex distribution of school-age children living in the 54 geographical sectors of the country, according to data from the National Census of 2001. A sample of 1,900 adolescents (11 to 18 year olds) was recruited. Adolescents filled in the questionnaire at school. A total of 1,194 (that is, 63% response rate) of self-reported questionnaires (40.07% boys) were returned. Inclusion criteria for the adolescents were to be between 11 and 18 years old, to be able to read and complete the questionnaires themselves, and to consent to be involved in the study. Ethical approval was attained from the National Ministry of Education. Previous research on the representativeness of the present sample has reported that non-responder interviews showed no significant differences between responders and non-responders with regard to the general perceived health of adolescents and parents, the marital status of parents, highest educational level, and type of residence, indicating that a selection bias is unlikely [13].

### Measures

The KIDSCREEN-52 questionnaire consists of 52 items and is based on a multidimensional HRQoL construct. The questionnaire was translated from English into Greek according to international guidelines using a backward-forward translation technique and international harmonization sessions [14,15]. The instrument assesses either the frequency of behavior/feelings or intensity of an attitude using a five-point Likert scale (1 = never, 2 = seldom, 3 = sometimes, 4 = often, 5 = always) and the recall period is 1 week. The questionnaire is categorized into ten dimensions: physical well-being, psychological well-being, moods and emotions, self-perception, autonomy, parent relation and home life, peers and social support, school environment, social acceptance (bullying) and financial resources. The score for each dimension was transformed to a 0 to 100 point scale with higher scores indicate better HRQoL.

The SDQ questionnaire contains 25 items, categorized into 5 scales of 5 items each: hyperactivity/inattention, emotional symptoms, conduct problems, peer problems and prosocial behavior [12]. Responses to each of the 25 items consisted of 3 options: not true, somewhat true, or certainly true. The version for youths was used in the present study, which was found to have satisfactory psychometric properties in Greek adolescents [16].

To assess familial socioeconomic status, the Family Affluence Scale (FAS) was used [17], addressing issues of family car ownership, children having their own unshared room, the number of computers at home and time the adolescents spent on holiday in the past 12 months. The FAS was collected from adolescents in seven categories (from 0 the lowest, to 7 the highest FAS category) and was recoded into three groups in the analysis (low FAS level (0 to 3), intermediate (4 to 5) and high FAS level (6 to 7)). The psychometric properties of the FAS are acceptable and support its use as a self-reported measure for adolescents [18,19]. Low FAS was expected to be associated with lower scores on KIDSCREEN-52 dimensions, but especially for financial resources.

To assess special chronic healthcare needs, the Children with Special Health Care Needs Screener (CSHCN) was included in the questionnaire, as a measure of physical and emotional chronic health status in children [20,21]. The CSHCN contains five question sequences: each question is followed by two additional questions, asking about the presence and duration of any health conditions. The five questions address the use or need of prescription medication; the use or need of medical, mental health or educational services; functional limitations; use and need of specialized therapies (occupational therapy, physiotherapy, speech therapy, and so on); and treatment or counseling for emotional or developmental problems, all associated with a health problem that has lasted or is expected to last 12 months or longer. It was expected that adolescents with a chronic health condition would have lower scores of HRQoL, especially for the dimension of Physical Well-Being [3].

### Statistical analysis

A confirmatory factor analysis (CFA) with maximum likelihood procedure was conducted in order to test how well the ten-dimension KIDSCREEN-52 model fits the data. The variance of the latent constructs was fixed at 1 during parameter estimation and the factors were allowed to be correlated. The fit of the CFA model was assessed using the χ^2 ^test, the comparative fit index (CFI), the goodness of fit index (GFI) and the root mean square error of approximation (RMSEA) [22]. For the CFI and GFI indices, values close to or greater than 0.95 are taken to reflect a good fit to the data [23]. RMSEA values of less than 0.05 indicate a good fit and values as high as 0.08 indicate a reasonable fit [23]. Also, a non-significant χ^2 ^statistic indicates a good fit, but χ^2 ^is usually sensitive to sample sizes and is usually significant for large sample sizes such as ours [22]. Scale internal consistency reliability was determined by calculation of the Cronbach α coefficient. Scales with reliabilities equal to or greater than 0.70 were considered acceptable. Validity was further examined with the correlation (Pearson's r) of KIDSCREEN-52 dimensions with SDQ scales. Correlation coefficients between 0.1 and 0.3 are considered low, between 0.31 and 0.5 moderate and over 0.5 high. Multivariate analysis of variance (MANOVA) was used in order to compare the scores of adolescents with chronic diseases with those of adolescents with no chronic diseases. Differences on KIDSCREEN-52 scales between the three socioeconomic status groups (low, middle and high) were also determined by the use of MANOVA. In order to control for multiple testing, Bonferroni correction was used. The *P *values reported are two-tailed. The statistical significance level was set at 0.05 and analysis was conducted using SPSS and AMOS (SPSS, Chicago, IL, USA) statistical software.

## Results

### Sample description

The total sample included 715 adolescent girls and 478 boys (N = 1,193). The total sample mean age was 13.6 (SD = 1.7). A total of 50.3% of participants belonged to the aged 11 to 14 group and 49.7% belonged to the aged 15 to 17 group. A total of 37.6% were identified in the low FAS group, 45% in the medium FAS group and 17.4% in the high FAS group. According to the CSHCN screener, 3.4% of the adolescents were suffering from a chronic health condition.

### Internal consistency reliability

Cronbach α and mean scale scores are presented in Table 1. All the scales of KIDSCREEN-52, exceeded the minimum reliability standard of 0.70. Cronbach α values ranged from 0.73 (bullying) to 0.90 (moods and emotions, social support and peers).

**Table 1:** Internal consistency and descriptive statistics of the KIDSCREEN-52 dimensions

	Mean	SD	Cronbach α
Physical well-being	66.1	19.2	0.83
Psychological well-being	70.0	19.3	0.88
Moods and emotions	72.6	18.2	0.90
Self-perception	66.4	21.0	0.85
Autonomy	58.7	23.5	0.81
Parent relation and home life	70.5	20.2	0.89
Peers and social support	70.4	21.3	0.90
School environment	64.2	18.7	0.88
Social acceptance (bullying)	91.9	14.0	0.73
Financial resources	69.5	24.3	0.89

### CFA results

As defined from the results of CFA the ten-dimensional model fitted the data well. The RMSEA, CFI and GFI values were 0.048, 0.971 and 0.965, respectively. None of the item cross loadings exceeded the item loadings on the intended latent construct. Estimates of the ten-dimensional model are shown in Table 2. Factor loadings were high and ranged from 0.61 to 0.88 indicating a strong association between the latent factors and their respective items (Table 2). The χ^2 ^test of the model was significant (χ^2 ^= 2,508.2, df = 1,299, *P *< 0.05).

**Table 2:** Factor loadings form the results of confirmatory factor analysis of the KIDSCREEN-52 study

Scale/item	b	Scale/item	b	Scale/item	b
Physical well-being:		Self-perception:		Social support and peers:	
How would you say your health is?	0.70	Have you been happy with way you are?	0.62	Have you spent time with your friends?	0.62
Have you felt fit and well?	0.68	Have you been happy with your clothes?	0.67	Done things with other girls and boys?	0.63
Been physically active?	0.61	Been worried about the way you look?	0.65	Have you had fun with your friends?	0.67
Been able to run well?	0.76	Felt jealous of way other girls/boys look?	0.67	You and your friends helped each other?	0.81
Felt full of energy?	0.67	Like to change something about your body?	0.74	Able to talk about everything with friends?	0.70
Psychological well-being:		Autonomy:		Have you been able to rely on your friends?	0.78
Has your life been enjoyable?	0.65	Have you had enough time for yourself?	0.64	School environment:	
Felt pleased that you are alive?	0.72	Able to do things/want to do in free time?	0.82	Have you been happy at school?	0.69
Felt satisfied with your life?	0.75	Had enough opportunity to be outside?	0.82	Have you got on well at school?	0.61
Been in a good mood?	0.79	Have you had enough time to meet friends?	0.72	Been satisfied with your teachers?	0.71
Felt cheerful?	0.72	Able to choose what to do in free time?	0.71	Have you been able to pay attention?	0.64
Had fun?	0.75	Parent relation and home life:		Have you enjoyed going to school?	0.61
Moods and emotions		Have your parent(s) understood you?	0.71	Got along well with your teachers?	0.64
Have you felt that you do everything badly?	0.64	Have you felt loved by your parent(s)?	0.72	Social acceptance (bullying)	
Have you felt sad?	0.62	Have you been happy at home?	0.67	Been afraid of other girls and boys?	0.70
Felt so bad that didn't want to do anything?	0.61	Parent(s) had enough time for you?	0.75	Have other girls and boys made fun of you?	0.72
Felt that everything in life goes wrong?	0.73	Have your parent(s) treated you fairly?	0.65	Have other girls and boys bullied you?	0.67
Have you felt fed up?	0.76	Able to talk to parent(s) when wanted to?	0.77	Financial resources	
Have you felt lonely?	0.77			Enough money to do things as friends?	0.84
Have you felt under pressure?	0.68			Had enough money for your expenses?	0.88
				Enough money to do things with friends?	0.86

### Convergent and discriminant validity

The associations of KIDSCREEN-52 dimensions with SDQ scales are shown in Table 3. Correlation analysis showed moderate to high coefficients for the expected relationships. The SDQ emotional symptoms subscale, as well as the total difficulties score correlated highest with the KIDSCREEN-52 moods and emotions dimension. Moderate correlations of the SDQ emotional subscale were also observed with the KIDSCREEN-52 dimensions of self-perception, psychological well-being, parent relation and home life, physical well-being and peers and social support. The SDQ hyperactivity subscale correlated highest with the KIDSCREEN-52 dimensions of school environment, moods and emotions and parent relation and home life. The SDQ peer problems subscale correlated highest with the KIDSCREEN-52 dimensions of peers and social support, moods and emotions and social acceptance (bullying).

**Table 3:** Correlation coefficients of the KIDSCREEN-52 dimensions with the Strengths and Difficulties Questionnaire (SDQ)

KIDSCREEN-52 dimensions	SDQ dimensions					
	
	Emotional symptoms	Conduct problems	Hyperactivity	Peer problems	Prosocial behavior	Total difficulties
Physical well-being	-0.33	-0.12	-0.23	-0.19	0.16	-0.31
Psychological well-being	-0.40	-0.11	-0.25	-0.27	0.16	-0.38
Moods and emotions	-0.58	-0.23	-0.38	-0.35	0.17	-0.56
Self-perception	-0.47	-0.18	-0.30	-0.25	0.17	-0.44
Autonomy	-0.28	-0.12	-0.12	-0.17	0.13	-0.22
Parent relation and home life	-0.34	-0.23	-0.31	-0.23	0.19	-0.38
Peers and social support	-0.31	-0.16	-0.12	-0.40	0.18	-0.30
School environment	-0.22	-0.21	-0.41	-0.18	0.29	-0.37
Social acceptance (bullying)	-0.26	-0.11	-0.16	-0.34	0.19	-0.29
Financial resources	-0.26	-0.20	-0.20	-0.25	0.11	-0.26

All correlations were significant at *P *< 0.001

Scores of KIDSCREEN-52 dimensions by FAS categories are shown in Table 4. There were no significant differences in scores between middle and high FAS groups except for physical well-being and financial resources. All scales except for social acceptance (bullying) and autonomy differed across FAS groups and lower scores were found for those belonging to low FAS category compared to those belonging to middle or high FAS category. Scores on self-perception were significantly different only between low and high FAS categories.

**Table 4:** Differences in KIDSCREEN-52 dimensions according to socioeconomic status as measured by Family Affluence Scale (FAS)

Dimension	Low FAS (A)		Middle FAS (B)		High FAS (C)		*P *value^a^	*P *value^b^		
			
	Mean	SD	Mean	SD	Mean	SD		A/B^b^	A/C^b^	B/C^b^
Physical well-being	62.8	19.2	66.7	18.7	70.6	18.1	< 0.001	0.004	< 0.001	0.033
Psychological well-being	67.2	19.7	71.6	18.5	70.5	20.0	0.006	0.002	0.041	0.692
Moods and emotions	69.6	18.8	74.1	17.4	73.9	18.7	0.003	0.002	0.010	0.868
Self-perception	64.1	21.3	67.1	20.8	68.5	21.2	0.020	0.072	0.007	0.174
Autonomy	57.2	24.0	59.5	24.0	58.7	21.6	0.112	0.138	0.252	0.635
Parent relation and home life	66.5	21.1	72.6	19.1	72.6	19.9	< 0.001	< 0.001	< 0.001	0.593
Peers and social support	67.5	22.3	71.3	20.2	72.4	21.1	0.029	0.027	0.023	0.558
School environment	61.5	19.1	65.7	18.2	66.4	18.2	0.007	0.007	0.009	0.604
Social acceptance (bullying)	91.6	14.4	92.1	13.7	91.8	13.9	0.917	0.881	0.774	0.677
Financial resources	60.1	25.4	73.4	21.6	81.1	19.8	< 0.001	< 0.001	< 0.001	< 0.001

^a^Multivariate analysis of variance (MANOVA).

^b^Post hoc analysis with Bonferroni correction for multiple comparisons.

Table 5 presents the mean scores of KIDSCREEN-52 dimensions for healthy adolescents and adolescents with chronic health condition. Healthy adolescents scored significantly higher on physical well-being, psychological well-being, school environment and social acceptance (bullying) dimensions.

**Table 5:** Comparison of KIDSCREEN-52 dimensions between chronically ill and healthy adolescents

Dimension	Healthy sample		Chronic health		
		
	Mean	SD	Mean	SD	*P *value^a^
Physical well-being	66.2	19.0	58.8	23.2	0.015
Psychological well-being	74.4	19.3	66.8	19.7	0.025
Moods and emotions	72.5	18.5	71.7	16.5	0.604
Self-perception	66.5	21.1	62.7	19.8	0.440
Autonomy	63.6	23.4	58.1	23.7	0.371
Parent relation and home life	70.3	20.0	67.4	24.2	0.295
Peers and social support	74.6	18.9	70.1	21.5	0.356
School environment	64.3	18.4	56.2	22.9	0.002
Social acceptance (bullying)	92.2	13.7	85.0	22.8	0.002
Financial resources	69.9	24.4	67.9	24.5	0.508

^a^Multivariate analysis of variance (MANOVA).

## Discussion

The KIDSCREEN-52 HRQoL questionnaire includes ten dimensions covering physical, psychological and social domains of quality of life. The main objective of the present study was to describe the psychometric properties of the Greek KIDSCREEN-52 in a sample of adolescents derived from schools. The analysis confirmed the dimension structure with sufficient psychometric properties.

The internal consistency reliability of the KIDSCREEN scales can be considered satisfactory with a Cronbach α coefficient of 0.73 or above for all dimensions. The internal consistency is similar to that reported from other studies referring to the reliability of KIDSCREEN [8-11].

There has been an increasing use of CFA for the exploration of psychometric properties of QoL questionnaires during recent years [24]. In the present study CFA confirmed the construct validity of the ten-dimensional measurement model a result consistent with other studies [8,10,11]. The RMSEA value was less than 0.05 and the CFI and GFI values were more than 0.95. The χ^2 ^test of the model was significant, but this can be explained since χ^2 ^statistics are sensitive to large sample sizes [22].

The convergent and discriminant validity analysis indicated that the KIDSCREEN-52 model showed a reasonable pattern of associations. With respect to the relationships between the generic HRQoL dimensions of the KIDSCREEN-52 and the mainly psychologically-oriented SDQ scales, it can be said that correlations between the two instruments were as predicted. The most significant correlations emerged in general between scales and dimensions tapping similar aspects of behavioral and emotional problems. For example, the correlation between the KIDSCREEN-52 moods and emotions dimension with SDQ emotional symptoms was -0.58 and the correlation between KIDSCREEN-52 peers and social support with SDQ peer problems was -0.40. Additionally, lower correlations between non-comparable scales support the construct validity in the form of discriminant validity (that is, low correlations were found between financial resources and SDQ scales).

High versus low FAS significantly differentiated adolescents in terms of all KIDSCREEN-52 dimensions except for autonomy and social acceptance (bullying). Previous studies have generally shown that HRQoL instruments are capable of discriminating between children and adolescents with different socioeconomic status [25].

The KIDSCREEN-52 questionnaire discriminated well in the hypothesized dimension of physical well-being between healthy adolescents and those with chronic physical or mental health problems. Also, differences in psychological well-being, school environment and social acceptance (bullying) were found between healthy adolescents and those with chronic health problems, but no differences between the aforementioned groups were found in the rest of the KIDSCREEN-52 dimensions. More research using the KIDSCREEN-52 questionnaire in clinical conditions is required in order to identify response patterns associated with those conditions.

### Strengths and limitations

The main strength of this study is its large and representative sample size. Also, the strength of our findings is that they are based on several goodness of fit criteria and associations of the measurement model.

However, there are several limitations to this study. Firstly, the identification of the group with chronic health problems was performed with a screening instrument, and no clinical information was available for physical and mental health problems. Thus, future studies should be provided in groups so that the severity of clinical conditions will be available.

Furthermore, because of the cross-sectional design of the study it was not possible to test the sensitivity of the KIDSCREEN-52 instrument to change. Changes over time should be evaluated with a longitudinal study design in future research. Pilot test in Spanish adolescents [26] has shown that KIDSCREEN follow up instrumentation seems adequate for collecting factors with potential influence on HRQoL.

## Conclusions

The results of the present study provide evidence that the KIDSCREEN-52 questionnaire seems to be a reliable and valid instrument for measuring HRQoL in Greek adolescents. The KIDSCREEN-52 questionnaire gives information for ten different aspects of HRQoL and it is also available in two shorter versions (the KIDSCREEN-27 index and KIDSCREEN-10 index) [27-29] and as proxy version for parents [30]. The KIDSCREEN questionnaire has been used in previous studies for clinical samples with obesity [31,32], cerebral palsy [33] and cancer [34]. Further research with different clinical samples should be conducted with appropriate cut-off points of HRQoL dimensions differentiating clinical from healthy samples. Measurement of HRQoL in adolescents could be useful in identifying populations at risk and provide targets for intervention in terms of public health.

## Competing interests

The authors declare that they have no competing interests.

## Authors' contributions

CT, AT, IZ and CD participated in the preparation of the paper. UR-S coordinated the European project 'Screening and Promotion for HRQoL in Children and Adolescents - A European Public Health Perspective'. YT had responsibility for overall supervision of the study. All authors read and approved the final manuscript.
